# *RMRP* Is a Non-Coding RNA Essential for Early Murine Development

**DOI:** 10.1371/journal.pone.0026270

**Published:** 2011-10-19

**Authors:** Joseph Rosenbluh, Deepak Nijhawan, Zhao Chen, Kwok-Kin Wong, Kenkichi Masutomi, William C. Hahn

**Affiliations:** 1 Department of Medical Oncology, Dana–Farber Cancer Institute and Departments of Medicine, Brigham and Women's Hospital and Harvard Medical School, Boston, Massachusetts, United States of America; 2 Broad Institute of Harvard and Massachusetts Institute of Technology (MIT), Cambridge, Massachusetts, United States of America; 3 Division of Cancer Stem Cell, National Cancer Center Research Institute, Tokyo, Japan; Childrens Hospital Los Angeles, United States of America

## Abstract

*RMRP* is a non-coding RNA that is ubiquitously expressed in both humans and mice. *RMRP* mutations that lead to decreased *RMRP* levels are found in the pleiotropic syndrome Cartilage Hair Hypoplasia. To assess the effects of deleting *RMRP*, we engineered a targeting vector that contains loxP sequences flanking *RMRP* and created hemizygous mice harboring this engineered allele (*RMRP* conditional). We found that insertion of this cassette suppressed *RMRP* expression, and we failed to obtain viable mice homozygous for the *RMRP* conditional allele. Furthermore, we were unable to obtain viable homozygous *RMRP* null mice, indicating that *RMRP* is essential for early embryonic development.

## Introduction

*RMRP* is a non-coding RNA that is highly expressed in a wide range of human and murine tissues [1]. Mutations in *RMRP* have been detected in individuals afflicted with Cartilage Hair Hypoplasia (CHH) [2], a syndrome characterized by short stature, sparse hair, immunodeficiency and in a subset of patients severe combined immunodeficiency or life threatening anemia. Cell-based reporter assays have shown that *RMRP* mutations result in decreased *RMRP* stability, which may account for the severe phenotypes seen in CHH [3]. Individuals that carry only a single *RMRP* mutation do not exhibit phenotypes associated with CHH [2]; however, affected individuals harbor mutations in both *RMRP* alleles [4] suggesting that these mutations inactivate the *RMRP* gene product.

The biological function(s) of *RMRP* remain incompletely understood. Biochemical studies have demonstrated that *RMRP* RNA binds to the mitochondrial posttranscriptional modification complex RNase MRP [5]. However, no apparent mitochondrial defects have been found in CHH patients. In addition, *RMRP* is also found in the nucleolus. We recently reported that together with the catalytic subunit of telomerase (hTERT), *RMRP* forms an RNA dependent RNA polymerase that converts single stranded *RMRP* RNA into double stranded *RMRP* [6].

To gain further insight into the biological functions of *RMRP*, we generated a genetically engineered mouse that lacks *RMRP*.

## Results and Discussion

**Figure 1:**
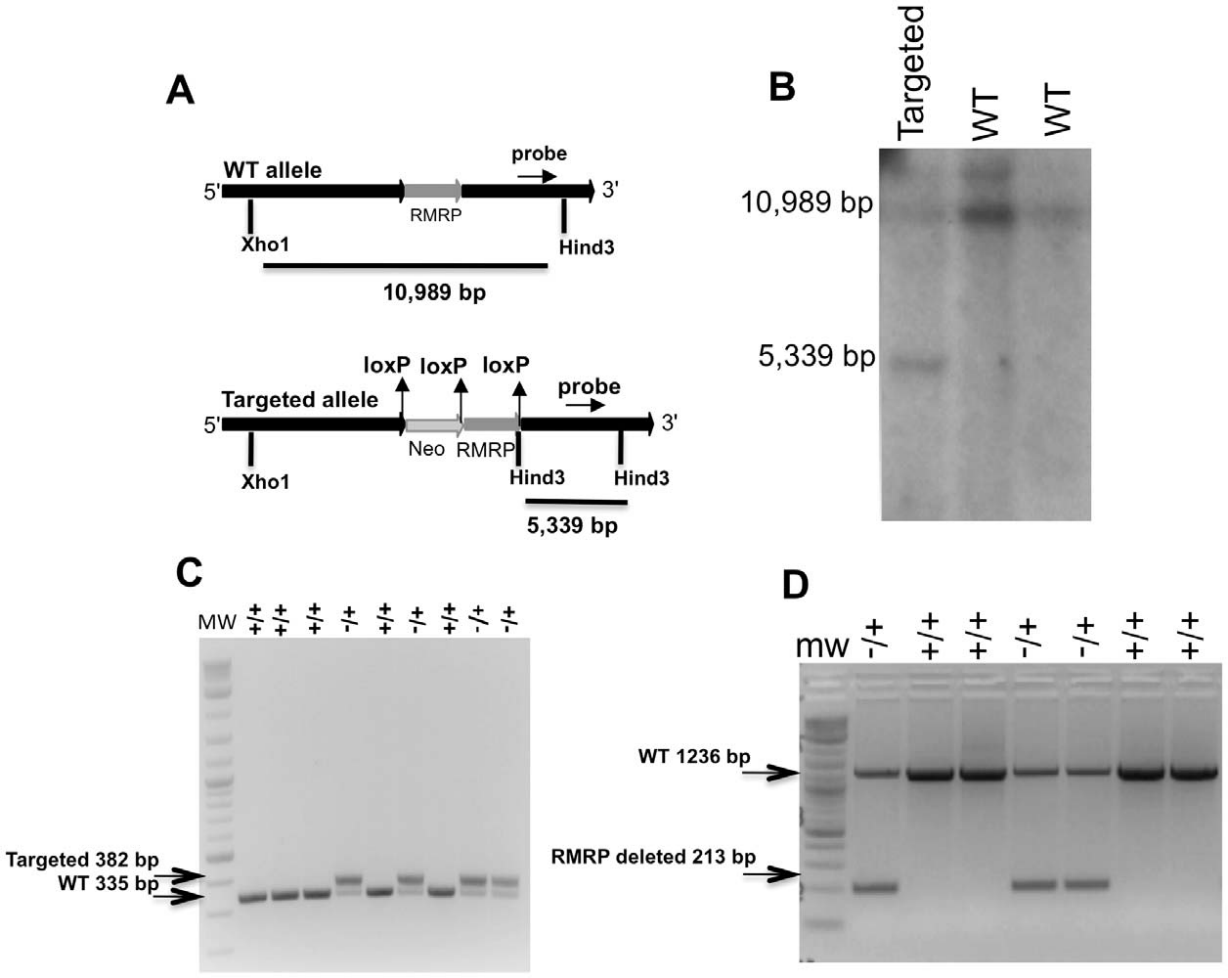
Targeting of murine *RMRP*. A. Murine targeting vector (MTV) B. Southern blot of ES cells following selection for alleles with integrated MTV (the southern probe is shown in figure 1a) C. PCR analysis of RC (*RMRP* conditional) pups D. PCR analysis of pups derived from the interbreeding of RC mice and mice expressing CMV-Cre.

We created a targeting vector specific for murine *RMRP* using the pEasyflox backbone [7]. The targeting vector contains the *RMRP* gene and promoter (800 bp up stream of murine *RMRP* [1]) flanked by two loxP sequences. A neomycin selectable marker flanked by two loxP sequences was placed upstream of *RMRP* (Figure 1A).

This targeting vector was introduced into mouse embryonic cells and individual clones containing the integrated targeting vector were selected by treatment with G418. Using southern blot analysis with a probe that can detect both the WT and targeted alleles, we found that 10% of the clones had integrated the *RMRP* targeting vector into the endogenous *RMRP* locus (Figure 1B). One of these clones was injected into female donor blastocysts producing 10 pups, 6 of which were chimeric, based on coat color. The chimeric mice were bred to FVB/N mice and the resulting pups were genotyped using a PCR based assay (Figure 1C). These mice contain the *RMRP* gene flanked by two loxP sequences and an insert coding for neomycin resistance upstream (*RMRP* conditional, RC) (Figure 1A).

We failed to obtain homozygous RC mice by crossing the hemizygous RC mice. Despite multiple attempts, we were unable to separate embryos earlier then E6.5 from the placenta. The RC mice harbor the neomycin resistance gene upstream of the *RMRP* gene, suggesting that insertion of DNA elements upstream of *RMRP* results in early embryonic lethality (Table 1). Thus, we hypothesized that the neomycin insertion impairs critical genomic elements that are essential for *RMRP* expression. Since prior work has confirmed that a subset of CHH patients harbor mutations in the *RMRP* promoter and these mutations decrease *RMRP* expression (1, 2), these observations suggest that the *RMRP* promoter is particularly sensitive to nucleotide changes.

**Table 1:** *RMRP* depletion is embryonic lethal.

Mating	N (male/female)		+/+	+/−	−/−
*RMRP* ^+/−^×*RMRP* ^+/−^	45 (20/25)	Expected	11.25	22.5	11.25
		Observed	19	26	0
RC^+/−^×RC^+/−^	47 (23/24)	Expected	11.75	23.5	11.75
		Observed	15	31	0

To confirm these findings, we tested whether complete deletion of *RMRP* would lead to a different phenotype. To this end, RC hemizygous mice were crossed to a mouse that ubiquitously and constitutively expresses the Cre recombinase (CMV-Cre). Using PCR with primers that are specific for the predicted engineered *RMRP* allele after recombination, we confirmed that the *RMRP* was deleted in the offspring of the hemizygous mice (Figure 1D). Similar to what we observed in RC mice, we failed to obtain pups harboring homozygous deletion of *RMRP* (Table 1). These observations suggest that that insertion of exogenous DNA sequences upstream of *RMRP* results in aberrant *RMRP* expression and results in embryonic lethality.

**Figure 2:**
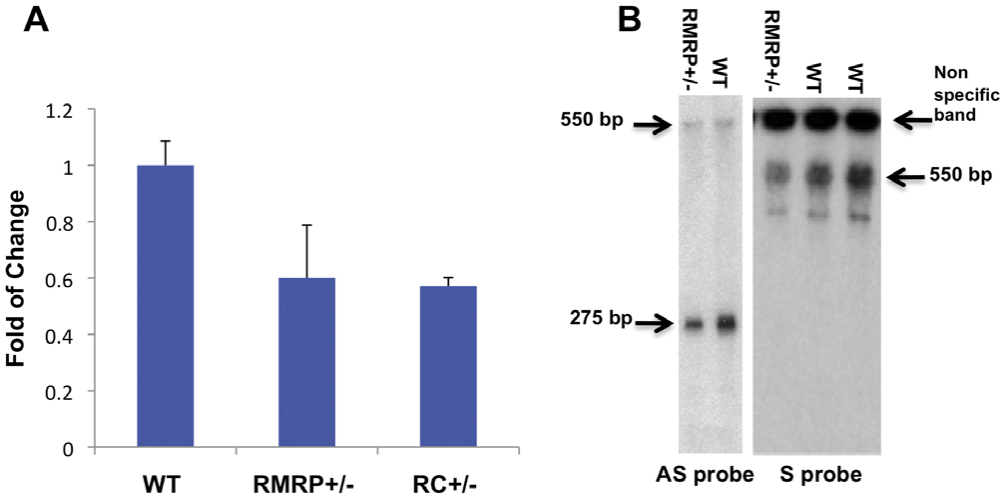
*RMRP* depletion leads to reduced levels of *RMRP* transcript. Total RNA was produced from E13.5 MEFs and *RMRP* level was measured by A. qRT-PCR B. Northern blot using either a sense or antisense *RMRP* probe. Error bars represent SD of three replicas.

The levels of *RMRP* may be critical for *RMRP* function. Specifically, Nakashima et al. have proposed a model by which *RMRP* mutations found in CHH patients leads to destabilization of *RMRP* [3]. When we assessed total levels of *RMRP* in murine embryonic fibroblasts (MEFs) obtained from *RMRP* or RC hemizygous mice, we found that *RMRP* was expressed at 50% of the level found in wild type MEFs (Figure 2A). RC and *RMRP* ^+/−^ mice were monitored from birth to 18 months of age and no abnormality in size, fur or behavior was detected. This is in consonance with what has been observed in human carriers of *RMRP* mutations [2].

We previously found that two species of *RMRP* are present in human cells: single stranded *RMRP* RNA and a double stranded *RMRP* RNA composed of a single RNA containing both the sense and antisense strands [6]. The double stranded version of *RMRP* requires the presence of the catalytic subunit of telomerase, TERT. Using Northern blot analysis with probes designed to detect sense or antisense *RMRP*, we detected decreased levels of both species of *RMRP* in total RNA extracted from *RMRP* ^+/−^ E13.5 MEFs as compared to WT MEFs (Figure 2B). The *RMRP* antisense probe detects both single and double stranded *RMRP* and the *RMRP* sense probe detects only double stranded *RMRP*. These observations demonstrate that reduction of *RMRP* reduces the function of the TERT-*RMRP* RdRP.

To assess the embryonic stage that requires *RMRP* we isolated embryos from breeding of *RMRP* ^+/−^ mice at several early time points. We failed to identify homozygous embryos as early as E6.5 (Table 2) indicating, that *RMRP* is required early in embryonic development.

**Table 2:** *RMRP* depletion is lethal in early stage embryos.

	N		+/+	+/−	−/−
E13.5	27	Expected	6.75	13.5	6.75
		Observed	11	16	0
E10.5	8	Expected	2	4	2
		Observed	1	7	0
E6.5	20	Expected	5	10	5
		Observed	4	16	0

**Figure 3:**
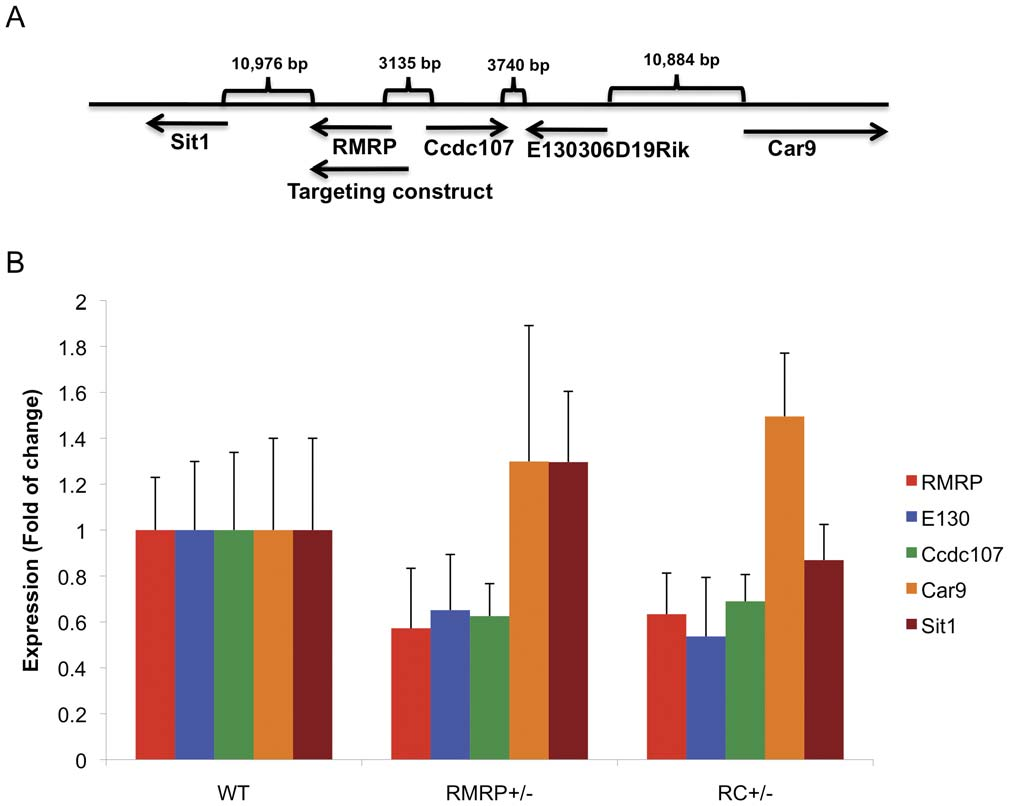
The effect of *RMRP* depletion on neighboring genes. A. Map of the genomic structure and genes surrounding *RMRP*. B. Expression of *RMRP*, *Sit1*, *Ccdc107*, *E130* and *Car9* in E13.5 MEFs from the indicated genotypes. Error bars represent SD of three replicas.

The *RMRP* gene is located in close proximity to several other genes including *Sit1*, *Ccdc107*, *E130* and *Car9* (Figure 3A). To assess whether the expression of these genes are affected by *RMRP* depletion, we measured the expression of these genes in E13.5 MEFs derived from WT, *RMRP* ^+/−^ or RC^+/−^ mice. We confirmed that *RMRP* levels were decreased by 50% in both *RMRP* ^+/−^ and RC^+/−^ MEFs. We also found that the expression of *Ccdc107* and *E130* were also decreased in *RMRP* ^+/−^ and RC^+/−^ MEFs while the expression of *Sit1* and *Car9* was not affected (Figure 3B). Although the targeting of *RMRP* involved the insertion of a small number of nucleotides, *RMRP*, *Ccdc107* and *E130* are located closely together, and deletion of *RMRP* affects the expression of all of these genes.

**Figure 4:**
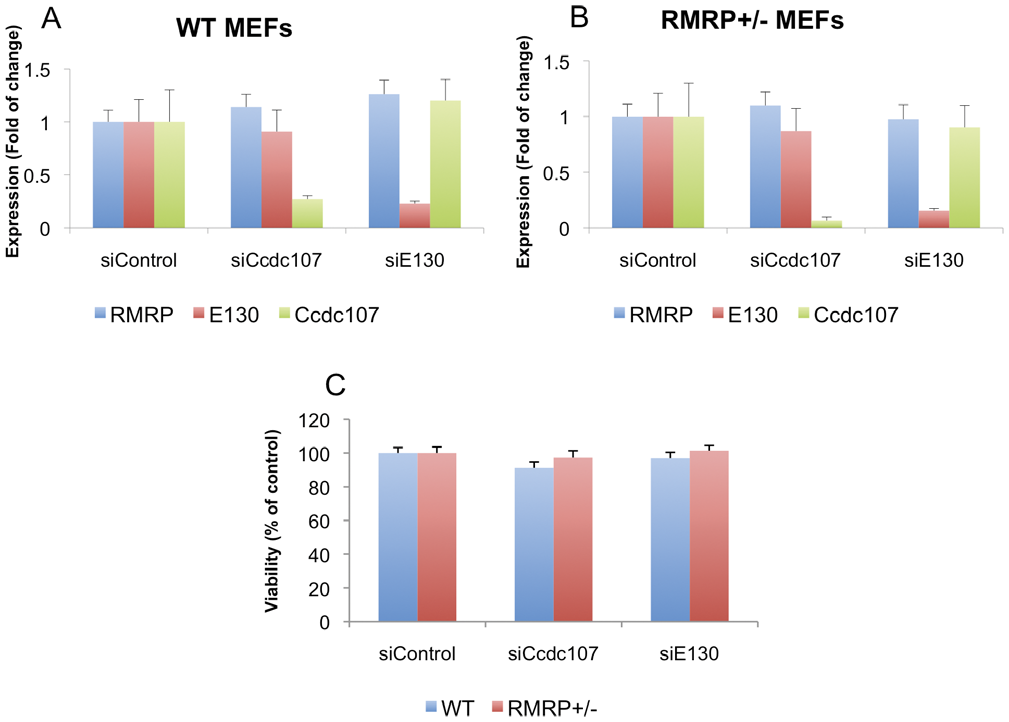
Genes near *RMRP* are not essential for cellular viability. MEFs from E13.5 mice of either A. WT or B. *RMRP*+/− were transfected with siRNAs targeting *Ccdc107* or *E130*. Three days later RNA was extracted from the cells and qRT-PCR was preformed using primers for *RMRP*, *Ccdc107* or *E130*. C. The same cells as in A and B were plated (5000 cells/well) in a 96 well plate and 7 days post transfection cell number was assessed by Cell titer glow. Error bars represent SD of three replicas.

Based on these observations, we tested whether *Ccdc107* or *E130* are essential for survival and thus may also contribute to the observed embryonic lethal phenotype. We used MEFs from E13.5 WT or *RMRP* ^+/−^ mice. Using RNAi, we reduced the expression of these genes to 5–20% of levels found in cells transfected with a control siRNA (Figure 4A and B). Seven days post transfection the cells were tested for viability, by monitoring the ATP content of the cells (Figure 4C). We failed to observe any alteration in cell proliferation/viability after suppression of either *Ccdc107* or *E130* indicating that these genes are not essential. However, we cannot eliminate the possibility that the residual low levels of *Ccdc107* or *E130* (5–20% of control) are enough to sustain viability and when completely deleted will lead to embryonic lethality. Due to the very close proximity between these genes it is very difficult to target *RMRP* without disrupting *Ccdc107* and *E130* expression and further attempts to target *RMRP* should take this into consideration.

*RMRP* is a ubiquitously expressed non-coding RNA [1] that has critical functions both in mice and humans. We found that *RMRP* is essential for development at early stages of embryogenesis. We further demonstrated that insertion of DNA elements upstream of the *RMRP* promoter cause a decrease in *RMRP* expression in hemizygous mice and are lethal in homozygous mice. These observations suggest that expression of *RMRP* is tightly regulated and essential for early developmental processes. We conclude that future attempts to target *RMRP* must consider the tight regulation and early requirement of *RMRP*.

## Materials and Methods

All laboratory animals will be cared for in the animal quarters of the Dana-Farber Cancer Institute under the direct supervision of the Dana-Farber Cancer Institute Animal Care and Use Committee (ACUC) under assurance number A3023-01. The Dana-Farber Cancer Institute is an Association for Assessment and Accreditation of Laboratory Animal Care International (AAALAC) accredited institution meeting or exceeding all standards for animal care and use. This work presented herein is has been approved by the ACUC under animal protocol 10-004.

### Construction of *RMRP* mouse targeting vector

The pEasyflox vector [7] was used as a backbone to create the murine targeting vector. A 1097 bp fragment corresponding to *RMRP* and 800 bp of the upstream promoter sequence was PCR amplified from RP23-207P5 (http://bacpac.chori.org) using Xba1-*RMRP* and *RMRP*-Sal1 primers (Table 3). The PCR product was digested with Xba1 and Sal1 and ligated to the same sites in the pEasyflox to create pEasyflox-*RMRP*. Next a 4064 bp fragment upstream of the *RMRP* gene was PCR amplified from RP23-207P5 using F5′Cla1 and R5′Not1 primers (Table 3). Following digestion with Not1 and Cla1 the PCR product was ligated to the same sites in pEasyflox-*RMRP* to create pEasyflox-5′-*RMRP*. The downstream sequence of the targeting vector was amplified from RP23-207P5 using F3′Hind3 and R3′Xho1 primers (Table 3) and the 4029 bp fragment was digested with Hind3 and Xho1 and ligated into the same sites in pEasyflox-5′-*RMRP* to create the *RMRP* mouse targeting vector. The sequence of the vector was confirmed by direct sequencing.

**Table 3:** primers used in this study.

Name	Seq (5→3)
Xba1-*RMRP*	ATATTCTAGATCCATGGGTGTTTTGTTCCCAAATC
*RMRP*-Sal1	ATATGTCGACGCAGCTCGCTCTGAAGGCCTGT
F5′Cla1	ATATATCGATGGATCCTGAAATTTGAAGGCAAATGGCAAATGG
R5′Not1	ATATGCGGCCGCAGGAGTTACAGAGACAAAGTTTGGAG
F3′Hind3	ATATAAGCTTCTTAGCTTCTAGGCGCGACTAATTT
R3′Xho1	ATATCTCGAGGGATCCGGGCCCAAAATATACTTGAAGTAGTT
F*RMRP*	TGCTGAAGGCCTGTATCCT
R*RMRP*	TGAGAATGAGCCCCGTGT
FRCgeno	TGAGCCCCGTGTGGTTGGTGC
RRCgeno	AGACCAATTTTCTCACCATAACCAAA
F*RMRP*geno	TTGCTAGTGTATGCAATGGTGTCAG
R*RMRP*geno	TTGTAGAGTCATAAATTAGTCGCGC
FSouthern	GATTTCCCATCTACTACTTACACTGA
RSouthern	CTGTCTCCCATGGAATGTACAGTGGCC
F*Ccdc107*	GGCACACCCAGAACGGGGCTC
R*Ccdc107*	CGCAGTCAGAGCAACAGCTGGT
F*E130*	CTGGTGGCCACGTACTTCCTGC
R*E130*	TACCCTTCAGGCGCAGCTGAAG
FSit1	TACAACTGTACCACTGACGATCA
RSit1	ATCCCCTCCATAAGCCACG
FCar9	CCGGAACTGAGCCTATCCAAC
RCar9	CAGGAATACCGGGCCTTGC

### Northern blot

RNA was extracted from MEFs using an RNeasy kit (Qiagene). 10 µl of sample buffer (95% formamide 5% bromophenolblue) was added to 5 µg of total RNA in a total volume of 20 µl. The samples were applied to a 6% TBE-urea acrylamide gel (invitrogen cat number: EC6865BOX) and then blotted onto a Hybond-N+membranes (GE Healthcare). Following cross-linking with a UV crosslinker the membrane was pre-incubated for 1 hour at 60°C with hybridization buffer (0.5 M NaHPO_4_ PH = 7.2, 1 mM EDTA, 7% SDS). A radiolabeled RNA probe corresponding to the full length sense or antisense murine *RMRP* was added and incubated with the membrane overnight at 60°C. Following 4 washes with 2×SSC the gel was exposed and visualized.

### Southern blot

DNA was prepared from ES cells targeted with the murine *RMRP* targeting vector using the Qiaamp kit (Qiagene). 10 µg of DNA was digested with Hind3, loaded onto a 1% agarose fomaldhyde gel and blotted onto a Hybond-N+membranes (GE Healthcare). Following UV crosslinking the membrane was pre-incubated for 1 hour at 65°C with hybridization buffer (0.5 M NaHPO_4_ PH = 7.2, 1 mM EDTA, 7% SDS). Next the probe was added to the blot and allowed to incubate overnight at 65°C. Following 3 washes with 1×SSC the membrane was exposed. The probe used for detection of targeted cells was generated by PCR amplification from a BAC clone (RP23-207P5 (http://bacpac.chori.org)) using FSouthern and RSouthern primers (Table 3).

### Quantitative RT-PCR

Total RNA extracted from MEFs was reverse transcribed using the advantage RT-PCR kit (Clontech). Following reverse transcription the quantity of the transcript was determined using specific primers (Table 3) and syber green PCR mix (applied biosystems).

### Genotyping

Tails were clipped from three week old mice and DNA was prepared. Genotyping was done *RMRP* null mice were genotyped using F*RMRP*geno and R*RMRP*geno (Table 3) and RC mice were genotyped using FRCgeno and RRCgeno (Table 3).

### siRNA transfection and viability assay

For silencing the expression of *Ccdc107* or *E130*, we used three independent siRNA duplexes targeting each gene. The siRNAs were purchased from IDT *Ccdc107* cat number: MMC.RNAI.N001037913.12.5, MMC.RNAI.N001037913.12.2, MMC.RNAI.N001037913.12.1 and *E130* cat number: MMC.RNAI.N001013377.12.8, MMC.RNAI.N001013377.12.3, MMC.RNAI.N001013377.12.1. For the negative control Ambion siRNA negative control 1 was used (cat number AM4635) 100,000 MEFs were plated in a 6 well plate and were transfected with 20 ng of the duplex mix. Three days later RNA was extracted from a fraction of the transfected MEFs and expression was assessed by qRT-PCR. The same cells were plated onto a 96 well plate (5000 cells/well, 4 wells/sample) and 7 days post transfection viability was measured using cell titer glow (Promega) 15 µl/well.
